# Aging impacts CD103^+^ CD8^+^ T cell presence and induction by dendritic cells in the genital tract

**DOI:** 10.1111/acel.12733

**Published:** 2018-02-18

**Authors:** Marta Rodriguez‐Garcia, Jared M. Fortier, Fiona D. Barr, Charles R. Wira

**Affiliations:** ^1^ Department of Microbiology and Immunology Geisel School of Medicine at Dartmouth Lebanon NH USA

**Keywords:** dendritic cells, female reproductive tract, menopause, resident memory T cells, sexually transmitted infections, TGF‐β

## Abstract

As women age, susceptibility to systemic and genital infections increases. Tissue‐resident memory T cells (TRMs) are CD103^+^ CD8^+^ long‐lived lymphocytes that provide critical mucosal immune protection. Mucosal dendritic cells (DCs) are known to induce CD103 expression on CD8^+^ T cells. While CD103^+^ CD8^+^ T cells are found throughout the female reproductive tract (FRT), the extent to which aging impacts their presence and induction by DCs remains unknown. Using hysterectomy tissues, we found that endometrial CD103^+^ CD8^+^ T cells were increased in postmenopausal compared to premenopausal women. Endometrial DCs from postmenopausal women were significantly more effective at inducing CD103 expression on allogeneic naïve CD8^+^ T cells than DCs from premenopausal women; CD103 upregulation was mediated through membrane‐bound TGFβ signaling. In contrast, cervical CD103^+^ T cells and DC numbers declined in postmenopausal women with age. Decreases in DCs correlated with decreased CD103^+^ T cells in endocervix, but not ectocervix. Our findings demonstrate a previously unrecognized compartmentalization of TRMs in the FRT of postmenopausal women, with loss of TRMs and DCs in the cervix with aging, and increased TRMs and DC induction capacity in the endometrium. These findings are relevant to understanding immune protection in the FRT and to the design of vaccines for women of all ages.

## 1 INTRODUCTION

Epidemiological studies show a global increase in the incidence of sexually transmitted infections (STIs), including new HIV infections, in older adults (CDC, 2016; Ghosh, Rodriguez‐Garcia & Wira, 2013). As people age, the systemic immune system undergoes progressive dysregulation that results in increased susceptibility to infections and malignancies and reduced responses to vaccination and increased general inflammation (Ghosh et al., 2013). However, the extent to which aging affects mucosal cell‐mediated immune protection in the female reproductive tract (FRT) is unknown and represents a critical gap in our knowledge for the development of preventive and/or treatment strategies against STIs for women of all ages.

Menopause is part of the aging process in women, which occurs at the mean age of 50 years, with premature menopause occurring in women younger than 40 years (Walker & Herndon, 2008). With menopause, sex hormone fluctuations decline and hormone levels remain low and constant (Givan et al., 1997; Wira, Rodriguez‐Garcia & Patel, 2015). Sex hormones regulate immune cell populations and function in the FRT throughout the menstrual cycle to prepare for implantation and pregnancy (Wira et al., 2015). In particular, T cells are tightly regulated in a compartmentalized manner to protect from infections without interfering with implantation (Erlebacher, 2013; Wira et al., 2015). However, after menopause, when pregnancy is no longer a primary function, endometrial T cells undergo phenotypical and functional changes, such as increased Th17 cell frequency or increased CD8^+^ T cell cytotoxic activity (Rodriguez‐Garcia, Barr, Crist, Fahey & Wira, 2014; White et al., 1997).

T cells are critical components of the adaptive immune system that provide specific long‐lasting protection against pathogens (Sallusto, Geginat & Lanzavecchia, 2004). Naïve T cells generated in the thymus migrate to lymphoid tissues throughout the body. Following antigen recognition, naïve T cells differentiate into effector and memory T cells. Tissue‐resident memory T cells (TRMs) are a subset of memory cells that remain in tissues and do not recirculate (Gebhardt et al., 2009; Mueller & Mackay, 2016). CD8^+^ TRMs can be identified by the expression of CD69 and CD103, the αE portion of the αEβ7 integrin that allows interactions with E‐cadherin expressed on epithelial cells (Rosato, Beura & Masopust, 2017). CD103^+^CD8^+^ T cells provide critical protection against viral infections due to privilege anatomical location within the epithelium, rapid local cytotoxic function, and induction of tissue antiviral state through the production of IFNγ and other pro‐inflammatory cytokines (Rosato et al., 2017; Schenkel et al., 2014).

In humans, memory T cells with TRM characteristics have been identified at multiple mucosal surfaces including the lung, the intestines, and the FRT (Thome & Farber, 2015; Thome et al., 2014). In the FRT, more than 95% of T cells are effector memory (Saba et al., 2010; Yeaman et al., 1997), with a high proportion of T cells expressing CD103 in the vagina, endocervix (CX), ectocervix (ECX), and endometrium (EM) (Duluc et al., 2013; Moylan et al., 2016; Rodriguez‐Garcia et al., 2017). Along with resident T cells, mucosal surfaces are populated by multiple subsets of resident DCs critical for the induction and maintenance of T‐cell responses (Schlitzer, McGovern & Ginhoux, 2015). DCs are known to be strongly influenced by the tissue environment, regarding their numbers, phenotype, and function (Schlitzer et al., 2015). We recently described a compartmentalized distribution of DCs in the FRT, with specific DC subsets restricted to the EM, while other subsets were distributed throughout the upper and lower FRT (Rodriguez‐Garcia et al., 2017). Studies with human DCs from the lung and vaginal mucosa demonstrate that certain DC subsets can induce CD103^+^ T cells with tissue‐resident characteristics (Duluc et al., 2013; Yu et al., 2013), suggesting that tissue DCs have the ability to control mucosal accumulation of TRMs.

While it is known that TRMs are present in the FRT, the extent to which aging influences their distribution and regulation in different anatomical locations within the FRT is unknown. Moreover, it is also unknown whether DCs from FRT sites other than the vagina have the ability to induce TRMs, and how aging and menopause impact their function. Given the epidemiological evidence for increased STIs in older women and the recognition that the FRT is a mucosal site with restricted access to T cells (Nakanishi, Lu, Gerard & Iwasaki, 2009), understanding the distribution, dynamics, and mechanisms for induction of TRMs is critical to unravel the underlying mechanisms that regulate immune protection in women of all ages.

Here, we report that menopausal status and aging control CD103^+^CD8^+^ T cell presence in a site‐specific manner in the FRT, through the modulation of DCs in a TGFβ signaling‐dependent mechanism. Our findings have broad implications, since they are relevant for explaining the increased risk of infection (STIs) in older women, immunological control of gynecological cancers, and to more fully understand the immunology of pregnancy.

## 2 RESULTS

### 2.1 Menopausal status and aging regulate the distribution of CD103^+^ T cells in the FRT

**Figure 1:**
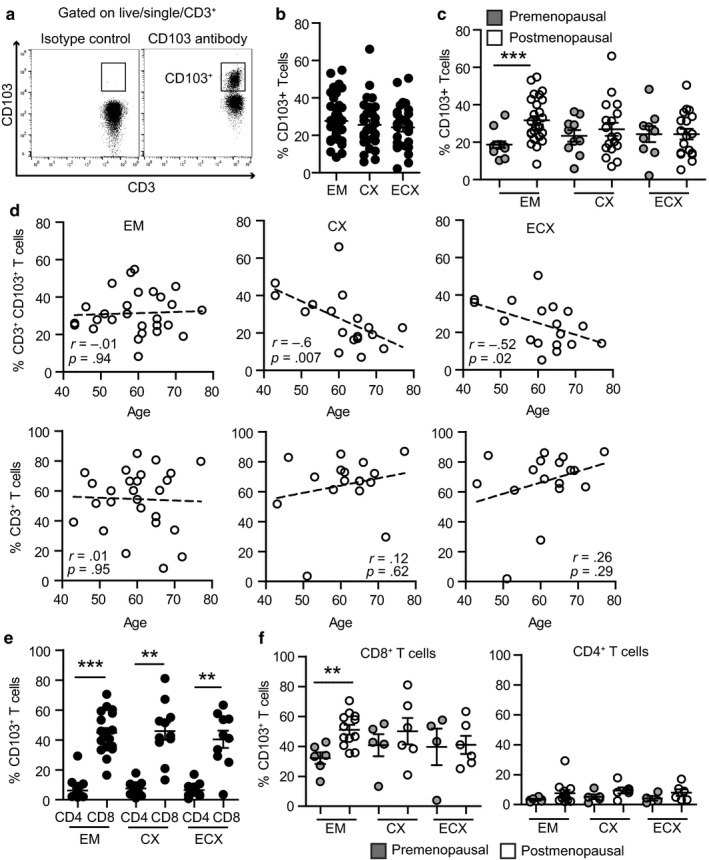
Menopausal status and aging differentially regulate CD103^+^ T cells in different sites of the female reproductive tract. (a) Representative plot of the gating strategy for CD103^+^ T cells within the CD3^+^ population. Left plot shows the isotype control and right plot the staining with anti‐CD103 antibody. (b) Percentage of CD103^+^ T cells within the CD3^+^ population in endometrium (EM = 40), endocervix (CX = 28), and ectocervix (ECX = 27) and (c) after separating pre‐ (gray; EM = 13, CX = 10, ECX = 9) and postmenopausal women (white; EM = 27, CX = 18, ECX = 18). Each dot represents a single patient. Mean ± *SEM* are shown; ***p < 0.001, nonparametric Kruskal–Wallis test with Dunn's post‐test correction for multiple comparisons. (d) Correlation between age and percentage of CD103^+^ T cells (top row) or percentage of CD3^+^ T cells (bottom row) in EM (*n* = 27), CX (*n* = 18), and ECX (*n* = 18) from postmenopausal women; Spearman correlation. (e) Percentage of CD103^+^ T cells within CD8^+^ and CD4^+^ T cells from EM (*n* = 18), CX (*n* = 11), and ECX (*n* = 10) and (f) after separating pre‐ and postmenopausal women. ***p < .001; **p < .01; Mann–Whitney *U*‐test

To define tissue distribution of resident T cells in the FRT, three different anatomical compartments (EM, CX, and ECX) were analyzed to determine what percentage of CD3^+^ T cells also expressed CD103 (Figure 1a and Figure S1a). Recognizing that resident T cells can be identified by the presence of CD69 and CD103 (Gebhardt et al., 2009), in preliminary studies we determined CD69 and CD103 expressions (Figure S1b). CD69 was widely expressed on FRT T cells, while CD103 expression was more restricted. Since CD103^+^ T cells co‐stained for CD69 (Figure S1b), consistent with previous reports (Posavad et al., 2017), we focused our studies on the CD103^+^ CD3^+^ T cell subset. Consistent with our previous observations (Rodriguez‐Garcia et al., 2017), CD103^+^ T cells were present at the three sites analyzed with no significant differences between upper and lower FRT (Figure 1b). To investigate potential changes after menopause in the percentage of CD3^+^ T cells that were CD103^+^, results from Figure 1b were stratified according to menopausal status (characteristics of the patients shown in Table 1). The percentage of CD3^+^CD103^+^ T cells was significantly increased in the EM from postmenopausal compared to premenopausal women (Figure 1c), with no changes observed in CX and ECX. These results are consistent with the CD103 mean fluorescence intensity (MFI) (Figure S1c), showing no significant differences between tissues, but increased CD103 MFI in the EM from postmenopausal compared to premenopausal women. No differences were found between the proliferative or secretory phases of the menstrual cycle in the premenopausal group (not shown). This differential distribution between pre‐ and postmenopausal women represents a new example of site‐specific changes in FRT T cells.

**Table 1:** Characteristics of the women included in the study

	Premenopausal	Postmenopausal
Number of women	21	30
Age median (range)	39 (27–48)	58 (46–77)
Menstrual stage		
Proliferative	60%	
Secretory	40%	
Atrophic		100%

Since menopause is linked to the aging process, we asked whether age could influence CD103^+^ T cells, by calculating the correlation coefficient between age and the percentage of CD3^+^ T cells that express CD103 for each tissue. Recognizing that sex hormones regulate T cells in premenopausal women (Wira et al., 2015), only postmenopausal women were included in this analysis to eliminate the confounding effects of sex hormones. Unexpectedly, we found a negative correlation with aging in the CX and ECX (Figure 1d, top row), while the percentage of CD3^+^CD103^+^ T cells remained unchanged in the EM. To determine whether this was a specific depletion of the CD103^+^ T cell subset, we also calculated the correlation coefficient between age and the percentage of total CD3^+^ T cells within the CD45^+^ immune cell population. As shown in Figure 1d (bottom row), no age‐dependent changes were detected in any of the tissues for the total CD3^+^ T cell population. These findings suggest that after menopause, as women age, CD103^+^ T cell presence progressively decreases in the cervix (CX and ECX), but remains unaltered in the EM.

Since CD103 can be expressed on CD4^+^ and CD8^+^ T cells (Rosato et al., 2017), we investigated which T cell subpopulation was expressing CD103 in FRT tissues in a subset of the patients shown in Figure 1b (gating strategy shown in Figure S1a). The majority of T cells expressing CD103 were CD8^+^ T cells (Figure 1e), with less than 10% of CD103^+^CD4^+^ T cells present. Importantly, menopausal status uniquely influenced CD103^+^CD8^+^ T cells, but not CD103^+^CD4^+^ T cells in the EM (Figure 1f). Consistent with the data presented in Figure 1c, no differences were found in CX and ECX between pre‐ and postmenopausal women for CD103^+^CD8^+^ or CD103^+^CD4^+^ T cells.

### 2.2 Endometrial DCs induce the expression of CD103 on naïve T cells

**Figure 2:**
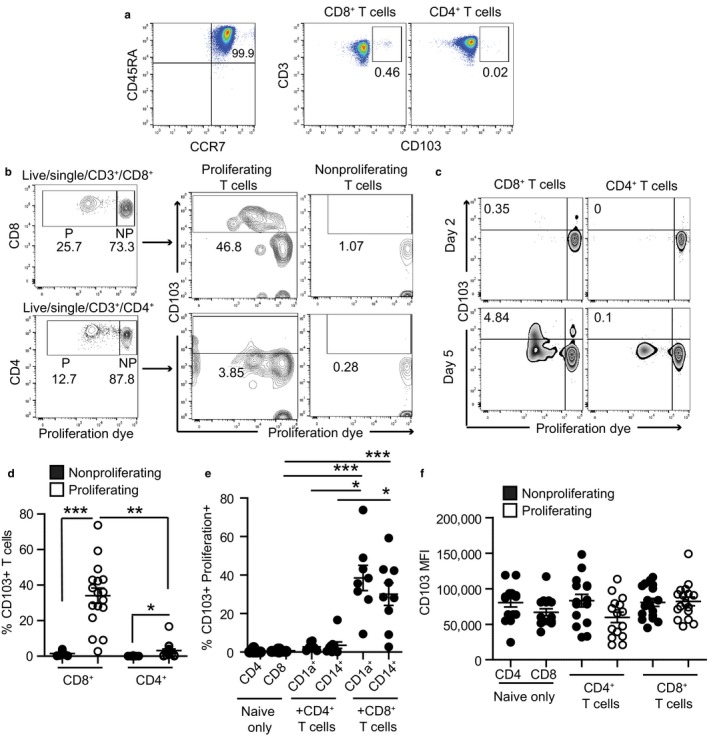
Endometrial DCs induce expression of CD103 on naïve T cells. (a) Representative plot of purity and CD103 expression on freshly isolated naïve T cells from blood before co‐culture (day 0). (b) Representative example of T‐cell proliferation rate and CD103 expression after allogeneic stimulation of naïve T cells with tissue DCs from EM. Upper row shows CD8^+^ T cells, and lower row shows CD4^+^ T cells. CD103 expression on proliferating (P, proliferation dye low) and nonproliferating (NP, proliferation dye high) cells is shown in middle and right panels, respectively. (c) Representative example of CD103 upregulation over time after allogeneic stimulation of naïve T cells with tissue DCs from EM. (d) Comparison of the percentage of CD103^+^ T cells within the proliferating or nonproliferating cell gates (*n* = 17). (e) Percentage of CD103^+^ T cells after allogeneic stimulation with EM DCs purified using CD1a^+^ (*n* = 8) or CD14^+^ (*n* = 9) magnetic bead selection. The graph shows proliferating CD103^+^ T cells, except in the naïve only control group, for which cells did not proliferate. (f) CD103 MFI (mean fluorescence intensity) on CD4^+^ CD103^+^ T cells (CD4^+^ T cells) or CD8^+^ CD103^+^T cells (CD8^+^ T cells) after allogeneic stimulation with EM DCs or naïve T cells cultured without DCs as a control (naïve only) (*n* = 17). *p < .05; **p < .01; ***p < .001; Kruskal–Wallis test with Dunn's post‐test correction for multiple comparisons

Human DCs from the lung have been reported to induce CD103^+^ T cells with TRMs characteristics (Yu et al., 2013). To evaluate whether FRT DCs regulate CD103^+^ T cells, DCs purified from the EM were tested for their ability to induce CD103 expression on allogeneic naïve T cells. DCs were purified using CD1a‐ or CD14‐positive selection as previously described (Rodriguez‐Garcia et al., 2017), to examine whether different FRT DC populations are functionally similar. Naïve T cells purified from blood expressed very low levels of CD103 at day 0 (Figure 2a), consistent with previous publications (Rosato et al., 2017). After 6 days in co‐culture, EM DCs increased the percentage of CD103^+^ cells on allogeneic naïve T cells that proliferated (Figure 2b, middle panels), but not on nonproliferating cells (Figure 2b); CD103 expression was induced in a time‐dependent manner (Figure 2c). The percentage of CD103^+^ T cells was significantly increased on proliferating CD8^+^ and CD4^+^ T cells compared to nonproliferating cells (Figure 2d). Furthermore, the percentage of CD103^+^ T cells was significantly higher for CD8^+^ T cells compared to CD4^+^ T cells (Figure 2d), consistent with the expression pattern found on freshly isolated T cells from FRT tissues (Figure 1e). Both CD1a^+^ and CD14^+^ selected DCs induced naïve T‐cell proliferation and increased the percentage of CD8^+^CD103^+^ T cells, with no detectable differences between CD1a^+^ and CD14^+^ selected DCs (Figure 2e). In contrast to the increase in the percentage of CD103^+^ T cells after proliferation, CD103 MFI did not change, and no differences were found between CD4^+^ and CD8^+^ T cells, whether they were cultured in the presence or absence of DCs (Figure 2f).

### 2.3 TGFβ signaling is responsible for the induction of CD103 expression on naïve CD8^+^ T cells

**Figure 3:**
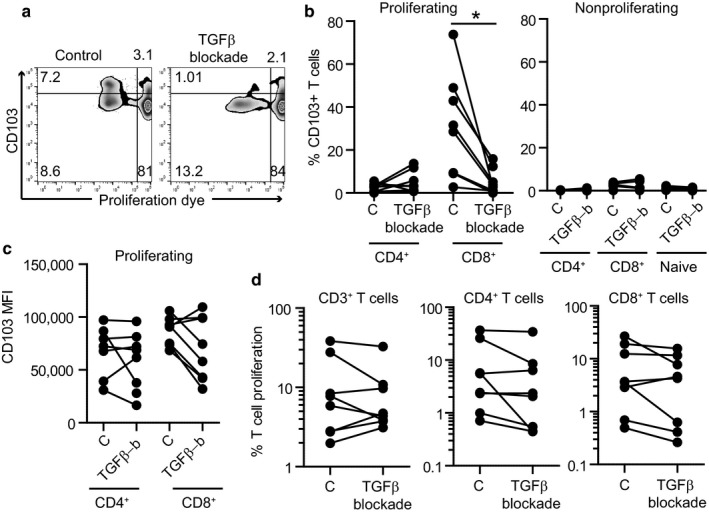
Endometrial DCs induce expression of CD103 on naïve CD8^+^ T cells through TGFβ signaling. (a) Representative plot of CD103 expression on proliferating and nonproliferating cells in the absence (control) or presence (TGFβ blockade) of the TGFβ signaling inhibitor SB431542. (b) Percentage of CD103 expression on proliferating (left) or nonproliferating (right) CD4^+^ and CD8^+^ T cells in the absence (C: control) or presence of the TGFβ signaling inhibitor SB431542 (TGFβ‐b) (*n* = 8). (c) CD103 mean fluorescence intensity on proliferating CD4^+^ and CD8^+^ T cells in the absence (C: control) or presence (TGFβ‐b) of the TGFβ signaling inhibitor SB431542 (*n* = 8). (d) Proliferation rate of total CD3^+^ T cells, CD4^+^ T cells, or CD8^+^ T cells after allogeneic stimulation with EM DCs in the presence of TGFβ signaling inhibitor (*n* = 8). Each dot represents a single patient. Mean ± *SEM* are shown. *p < .05; Wilcoxon‐matched pair test

Expression of CD103 exclusively on proliferating T cells in the presence of genital DCs (Figure 2d,e) suggested that induction of expression was mediated through DC‐T cell interactions rather than soluble factors. Recognizing that lung DCs can upregulate CD103 on T cells through membrane‐bound TGFβ signaling (Yu et al., 2013), we tested the hypothesis that DC membrane‐bound TGFβ was responsible for regulating CD103^+^ T cells in the FRT. DC‐naïve T‐cell co‐cultures were incubated with SB431542, a known inhibitor of TGFβ signaling (Ochiel, Ghosh, Fahey, Guyre & Wira, 2010). As shown in a representative example in Figure 3a, TGFβ signaling inhibitor selectively prevented CD103 expression on proliferating CD8^+^T cells, but it had no effect on overall T‐cell proliferation. When SB431542 was present, proliferating CD103^+^ T cells were markedly reduced, but the CD103^−^ T cell population was increased (Figure 3a, right plot), suggesting that the induction of CD103 expression was selectively inhibited during the proliferation process. The significant reduction in CD103^+^CD8^+^ T cells in the proliferating population observed after treatment with SB431542 (Figure 3b) was not mimicked in CD4^+^ T cells, even though a small but significant increase in CD103‐expressing cells was observed in proliferating CD4^+^ T cells (Figure 2c). As a control, no changes in CD103 expression were observed in nonproliferating cells or naïve T cells cultured in the absence of DCs when SB431542 was present (Figure 3b, right graph). CD103 MFI remained unchanged in the presence of the TGFβ signaling inhibitor (Figure 3c), consistent with the lack of changes in CD103 MFI observed during proliferation (Figure 2f). Importantly, the decrease in CD103^+^ T cells after proliferation in the presence of SB431542 was not a consequence of reduced proliferation, as demonstrated by the lack of changes in percentage of proliferating T cells (Figure 3d).

Overall, these results demonstrate a selective regulation by DCs of the induction of CD103 expression on CD8^+^ T cells through the TGFβ signaling pathway.

### 2.4 Menopausal status regulates induction of CD103^+^ T cells by endometrial DCs

**Figure 4:**
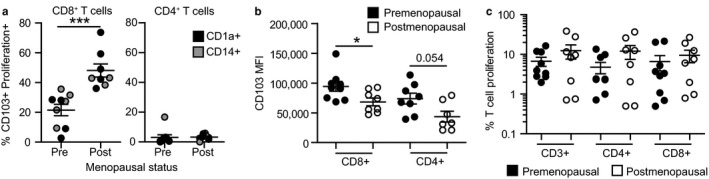
Menopausal status regulates endometrial DC ability to induce CD103 expression on CD8^+^ T cells. (a) CD103^+^ T cell percentage, (b) CD103 mean fluorescence intensity, and (c) proliferation rate after allogeneic stimulation of naïve T cells with EM DCs from pre‐ (*n* = 9) or postmenopausal (*n* = 8) women. Results from CD1a^+^ and CD14^+^ DCs are shown combined. ***p < .001; *p < .05; Mann–Whitney *U*‐test

Next, we asked whether menopausal status influences the ability of endometrial DCs to induce CD103^+^ T cells. For this purpose, we reanalyzed the data from Figure 2d based on menopausal status. Since no differences were observed between CD1a^+^ or CD14^+^ selected DCs, results from these two subsets were pooled to increase statistical power. After allogeneic co‐culture of the same number of endometrial DCs and blood‐derived naïve T cells (see methods), DCs from premenopausal women were less effective than DCs from postmenopausal women at inducing CD103 expression selectively on CD8^+^ T cells (Figure 4a). In contrast, an unexpected reduction in CD103 MFI was detected in postmenopausal women, both on CD8^+^ and on CD4^+^ T cells (Figure 4b). Recognizing that CD103^+^ T cells from postmenopausal women had increased CD103 MFI (Figure S1c), our findings suggest that DCs control the expression of CD103 on T cells; however, it does not exclude the possibility that additional tissue factors also modulate CD103 expression on CD8^+^ T cells. Importantly, induction of naïve T‐cell proliferation was unaffected by menopausal status (Figure 4c), demonstrating selective regulation of specific DC functions.

### 2.5 Progressive general decline in DC numbers throughout the FRT, but selective decline in CD103^+^ T cells in the cervix with aging

**Figure 5:**
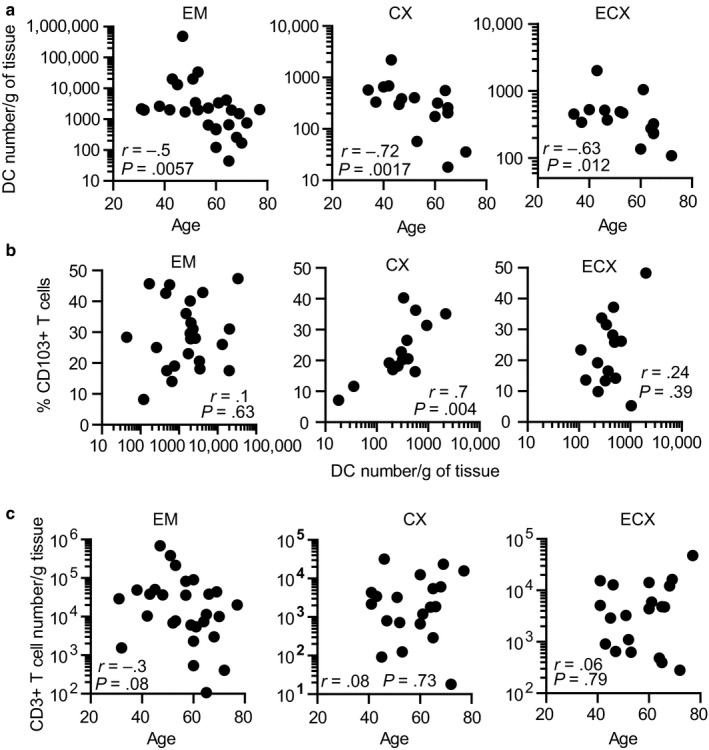
Dendritic cells (DC) numbers and CD103^+^ T cell percentage decline in cervix with aging. (a) Correlation between DC number and age (EM = 27; CX = 16; ECX = 15) and (b) DC number and CD103^+^ T cell percentage (EM = 25; CX = 15; ECX = 15). (c) Correlation between age and total CD3^+^ T cells per gram of tissue (EM = 28, CX = 20, ECX = 20). Each dot represents a single patient; Spearman correlation

Next, we investigated whether DCs might be responsible for the progressive decrease in CD103^+^ T cells in CX and ECX after menopause (Figure 1d). Because we observed that DCs from CX and ECX of older women could not be isolated in sufficient numbers to perform proliferation assays, we quantified DC numbers and CD103^+^ T cell percentage in the same tissues to unmask any correlations with aging (gating strategy shown in Figure S1a). Figure 5a shows a significant progressive decrease in DC numbers as a function of age in the EM, CX, and ECX. Additionally, we found that DC number and CD103^+^ T cell percentage positively correlated in the CX (Figure 5b), but found no correlation in the EM or ECX. Recognizing that the decrease in total DC numbers could be a consequence of tissue atrophy with age, we quantified the absolute number of CD3^+^ T cells per gram of tissue, to understand whether decreases in cell numbers as a function of age is a general characteristic for all cell types in the FRT. As shown in Figure 5c, total numbers of CD3^+^ T cells were not affected by age, increasing the relevance of the decline in specific cell subsets.

These results indicate that aging is a critical regulator of DC number throughout the FRT and suggest that while DC number and CD103^+^ T cell percentage are associated in the CX, additional tissue‐specific factors may be involved in the EM and ECX.

## 3 DISCUSSION

Our study demonstrates the novel compartmentalization and regulation of CD103^+^ T cells and DCs in the FRT before and after menopause. Because women have a uniquely long postreproductive survival potential (Walker & Herndon, 2008), human studies are critical to unravel immunological changes induced by menopause and aging. Recognizing that human and mouse studies indicate that anatomical location influences T‐cell immunity, the studies presented here focus on tissues from three distinct anatomical sites in the FRT, from women between 27 and 77 years old, to define the extent to which CD103^+^ T cells and DCs are uniquely regulated in the FRT over time.

Our findings demonstrate an age‐dependent progressive reduction in TRMs in the CX and ECX after menopause. In contrast to the decline in cervix, we found that the percentage of TRMs increase in endometrial tissues of postmenopausal compared to premenopausal women and that this increase remains stable with postmenopausal aging. These findings were unexpected, since previous reports indicated no changes in TRM frequency with aging in other tissues, including the lung and intestines (Thome et al., 2014). Our findings suggest that differential mechanisms control the presence and the induction of TRMs in the FRT. The extent to which the differential effects of aging and menopausal status on TRM distribution between the EM and cervix are due to changes in induction by DCs or in tissue survival (Moreau et al., 2017) deserves further investigation.

Previous studies in the lung and the vagina have shown that subsets of DCs have the ability to induce CD103 expression on CD8^+^ T cells (Duluc et al., 2013; Yu et al., 2013), suggesting that CD103 induction is an “intrinsic” function of DCs (Yu et al., 2013). Our findings add to this information by demonstrating that endometrial DCs also induce CD103 expression on CD8^+^ T cells. However, an unexpected and novel observation from our study is that endometrial DCs’ intrinsic ability to induce CD103^+^ CD8^+^ T cells is regulated by local changes in the FRT with menopause. The implications of these findings are that TRM presence in the EM before and after menopause may be controlled through the regulation of DC function. Importantly, while DC's ability to upregulate CD103 was affected by menopausal status, their ability to induce T‐cell proliferation was not, suggesting a very selective regulation of DC function. The selectivity of this mechanism is further supported by our previously reported lack of changes in DC phenotype and HIV‐capture potential between pre‐ and postmenopausal women (Rodriguez‐Garcia et al., 2017). While we did prove the ability of endometrial DCs to induce the expression of CD103 on naïve T cells, whether DCs have the ability to modify CD103 expression levels on tissue memory T cells remains to be investigated.

From a mechanistic standpoint, others have shown that TGFβ production in certain tissues promotes CD103 expression, either soluble TGFβ or membrane‐bound TGFβ (Casey et al., 2012; Lee et al., 2011; Mackay et al., 2013; Yu et al., 2013). Our studies build on this foundation to demonstrate that signaling through TGFβ bound to the membrane of endometrial DCs was responsible for the induction of CD103 on naïve CD8^+^ T cells. Future studies will explore the extent to which changes in DC membrane‐bound TGFβ are involved in the differential ability of pre‐ and postmenopausal DCs to elicit CD103 expression on T cells. At this point, the mechanisms responsible for the selective TGFβ signaling effects on CD8^+^ T cells but not on CD4^+^ T cells remain unclear; our findings of selective differential effects in the human FRT extend previously reported findings of selective effects on survival of CD8^+^ and CD4^+^ T cells in mice (McKarns & Schwartz, 2005). It also remains unclear whether the same mechanisms are responsible for the induction of CD103^+^ T cells in the CX and ECX. While we speculate the DCs from CX and ECX also induce CD103 expression on T cells, based on our preliminary results and previous publications of others with vaginal DCs (Duluc et al., 2013), whether upregulation at all sites in the FRT is mediated through TGFβ signaling needs to be investigated, given the evidence in mice that nasal TRM induction was TGFβ‐independent, while lung TRM induction was TGFβ‐dependent (Pizzolla et al., 2017). Furthermore, whether DC function in the FRT is regulated in response to the presence or lack thereof of sex hormones and/or the local tissue environment remains to be determined.

Our findings also demonstrate that aging is a critical factor in determining DC numbers in the FRT. However, whereas declining DC numbers and CD103^+^ T cell percentage with aging correlated in the CX in postmenopausal women, no correlation was found in the ECX or the EM. These findings suggest that distinct tissue‐specific factors regulate the presence and sustainability of CD103^+^ T cells in the different anatomical compartments of the FRT. Interestingly, the extracellular matrix has been described to change with age, including increased collagen stiffness, which may impair immune cell motility and survival (Moreau et al., 2017). While we observed a decrease in DCs and TRMs with age, no changes were observed for total T cell numbers per g of tissue, suggesting that specific immune cell subsets are more susceptible than others to age‐related changes in the FRT. It is possible that those cells that establish long‐term residence in tissues, such as TRMs or DCs, are preferentially affected by age‐related changes in extracellular matrix composition with age. Beyond the potential role of the extracellular matrix, a recent study demonstrated that exogenous fatty acids were necessary for mice TRM survival in the skin (Pan et al., 2017). Future studies are needed to identify those factors, whether soluble or extracellular matrix related, that influence the human FRT tissue environment to preserve CD103^+^ T cell survival. Whether other immune cell types present in the FRT, such as NK cells or macrophages, also decrease with age remains to be elucidated.

Site‐specific regulation of TRMs and DCs in the FRT is consistent with the contribution of each site to the reproductive function. We propose that, in premenopausal women, TRM presence is actively suppressed in the EM but not in the cervix, possible through a TGFβ‐dependent mechanism as a consequence of direct or indirect effects of sex hormones on endometrial DCs. Since the EM is the site for implantation, the regulation of TRMs with cytotoxic potential would be essential for successful pregnancy. In line with this hypothesis, a recent study found decreased expression of the tissue residency marker CD69 on CD8^+^ T cell populations in women with recurrent miscarriage when compared to controls (Southcombe et al., 2017). Whether implantation failure can be triggered by alterations in the resident T‐cell populations identified in our study remains to be determined. In contrast to the EM, CD103^+^ T cells are abundant in the cervix from premenopausal women, consistent with higher exposure to pathogens and the need for protection from potential ascending pathogens (Posavad et al., 2017). Our findings are in agreement with our previous demonstration of increased CD8^+^ T cell cytotoxic activity in the cervix of premenopausal women when compared to the endometrium (White et al., 1997) and with mouse models demonstrating that cytotoxic T cells and DC function are regulated to prevent rejection during pregnancy (Erlebacher, 2013).

In postmenopausal women, we observed a progressive decline in TRMs in the cervix, possibly due to decreased numbers of DCs in the endocervix and/or unidentified tissue‐specific factors in the ectocervix. In contrast to the cervix, TRMs increased in the EM following menopause, even though DC numbers declined with age. This could be due to an increased functional capacity of postmenopausal DCs as a consequence of the loss of suppressive mechanisms of DC function once pregnancy is no longer a primary function and possibly to offset the age‐dependent loss of immune protection in the lower FRT. For example, others have shown that pelvic inflammatory disease, caused by ascending pathogens into the uterus and fallopian tubes, is rarely diagnosed in postmenopausal women (Jackson & Soper, 1999). Our results might offer a possible explanation for these observations, namely that TRM immune protection, possibly mediated by DCs, is enhanced in the uterus to counter any increase in upstream pathogens owing to altered cervical protection.

Our observation of lower numbers of CD103^+^ T cells in the CX and ECX in older women correlates with epidemiological findings of increased susceptibility to certain STIs after menopause (Ghosh et al., 2013). Epidemiological studies demonstrate a second peak in the prevalence of human papillomavirus (HPV), a cause of cervical cancer, in women older than 45 years old (Gonzalez et al., 2010). Since TRMs are implicated in HPV infection control (Cuburu et al., 2012; Gravitt, 2012), our finding of a decrease in cervical TRMs with aging suggests an explanation for increased HPV infection rates in older women. Our studies are consistent with our previous findings that CD8^+^ T cell cytotoxic activity in the EM is low in premenopausal women but markedly elevated following menopause (White et al., 1997).

Our results are relevant to the future development of vaccines, since most genital pathogens represent an enormous challenge for developing vaccines that can induce genital immunity to prevent acquisition and transmission. Recent murine studies demonstrate that memory CD103^+^CD8^+^ T cells in the nasal mucosa prevent dissemination of pulmonary influenza virus infection to the lungs (Pizzolla et al., 2017). Animal studies have also demonstrated that TRMs mediate local protection against genital HSV‐2 infections (Shin, Kumamoto, Gopinath & Iwasaki, 2016). Therefore, induced immune cell protection against STIs in humans would likely require a potent resident memory T‐cell population (Iwasaki, 2010). Based on our findings, we speculate that despite vaccination, TRMs will not be equally induced and distributed throughout the FRT and will differ depending on women's age. For example, TRM endometrial protection might be impaired in younger women (premenopausal), while cervical protection will be suboptimal in older women (postmenopausal). As women age and remain sexually active, protection of the FRT at all ages is essential. Our findings are particularly relevant to HIV prevention, because most CD8^+^ T cell studies focus on the evaluation of blood T‐cell responses, which do not correlate with the protective effects of resident T cells in the FRT (Shin et al., 2016; White et al., 2001).

In conclusion, we demonstrate that menopausal status and aging influence CD103^+^CD8^+^ T cell induction and presence in the FRT. Our findings offer a novel perspective to understanding immune protection in the FRT and are relevant to STI infection risk, mucosal protection after vaccination, immunology of pregnancy, and tumor protection. The tissue‐specific factors that control TRM presence as well as DC function and presence in the FRT need to be identified to achieve effective mucosal protection throughout women's live cycle.

## 4 EXPERIMENTAL PROCEDURES

### 4.1 Study approval

Studies were approved by Dartmouth College Institutional Review Board and the Committee for the Protection of Human Subjects (CPHS). Written informed consent was obtained before surgery from HIV‐negative women undergoing hysterectomies at Dartmouth‐Hitchcock Medical Center (Lebanon, NH, USA). Surgery was performed to treat benign conditions including fibroids, prolapse, and menorrhagia. Hormonal contraceptives were not administered before surgery. Trained pathologists selected tissue samples from endometrium (EM), endocervix (CX), and ectocervix (ECX) free of pathological lesions and distant from the sites of pathology. Women were HIV‐ and HPV‐ but no additional information regarding other genital infections was available. Table 1 shows the characteristics of the women included in the study.

### 4.2 Tissue processing

Matched tissues from the endometrium (EM), endocervix (CX), and ectocervix (ECX) of the same patient were used whenever possible. In some cases, only endometrial tissue was provided by pathology. Vaginal tissues were not available. Tissues were processed to obtain a stromal cell suspension as described previously (Rodriguez‐Garcia et al., 2017), using 0.05% collagenase type IV (Sigma‐Aldrich, St. Louis, MO, USA) and 0.01% DNAse (Worthington Biochemical, Lakewood, NJ, USA). After filtering through a 20‐μm mesh screen (Small Parts) to separate epithelial cells from stromal cells, stromal cells underwent dead cell removal (Dead Cell Removal Kit, Miltenyi Biotec) as described (Rodriguez‐Garcia et al., 2014), resulting in more than 90% cell viability by trypan blue staining. After dead cell removal, mixed cell suspensions were used for phenotypical analyses by flow cytometry or further processed for cell isolation.

### 4.3 Flow cytometry

Mixed cell suspensions were stained for surface markers with combinations of the following antibodies: CD45‐vioblue 450, CD8‐FITC, CD19‐APC (Tonbo, San Diego, CA), HLA‐DR‐FITC, CD3‐viogreen, CD103‐PE (Miltenyi Biotec, Auburn, CA, USA), CD11c‐PerCp‐Cy5.5, CD69‐PE‐Cy7 (Biolegend, San Diego, CA), CD103‐PE‐Cy7, CD4‐PE (eBiosciences, San Diego, CA, USA), CD56‐APC (BD Pharmingen, San Diego, CA, USA). Dead cells were excluded with 7AAD (Southern Biotech) or zombie dye yellow staining (Biolegend). Analysis was performed on 8‐color MACSQuant 10 (Miltenyi biotech) or Gallios (Beckman Coulter) flow cytometers and data were analyzed with FlowJo software (Tree Star, Inc. Ashland, OR, USA). Expression of surface markers is shown as percentage of positive cells and MFI. Fluorescence minus one (FMO) strategy was used to establish appropriate gates. Comparisons between FMO and isotype controls showed no differences. For tissue dendritic cell (DC) quantification, DCs were identified using flow cytometry as CD45^+^, CD3‐, CD19‐, CD56‐, HLA‐DR^high^, CD11c^+^ cells as described before (Rodriguez‐Garcia et al., 2017), and the cell number was normalized to tissue weight.

### 4.4 CD14^+^ and CD1a^+^ cell isolation

Mixed cell suspensions were centrifuged by standard Ficoll gradient as described previously (Rodriguez‐Garcia et al., 2017), prior to DC isolation using positive magnetic bead selection with either the CD14^+^ or CD1a^+^ isolation kits (Miltenyi Biotec) according to the manufacturer's instructions. After two rounds of positive selection, purity of the CD14^+^ and CD1a^+^ population was about 90% (Rodriguez‐Garcia et al., 2017). Isolated DCs were plated in round‐bottom ultra‐low attachment 96‐well plates (Corning, Corning, NY, USA) in Xvivo15 (Invitrogen) supplemented with 10% human AB serum (Valley Biomedical) for in vitro allogeneic stimulation.

### 4.5 Allogeneic naïve T cell stimulation assay

Naïve T cells were purified from cryopreserved peripheral blood mononuclear cells (PBMCs) using the Naïve Pan T Cell Isolation Kit (Miltenyi Biotec). After purification, isolated naïve T cells were >99% CCR7^+^ CD45A^+^ as determined by flow cytometry with CD3‐APC‐Cy7 (Tonbo), CCR7‐PE‐Cy7 (Miltenyi Biotec), and CD45RA‐APC (Biolegend). Naïve T cells were stained with Cell Proliferation Dye eFluor‐670 (eBioscience) as recommended by the manufacturer. Purified mucosal CD1a^+^ or CD14^+^ cells (5 × 10^3^ cells) were plated with naïve T cells (7.5 × 10^4^ cells) (1:15 ratio) in round‐bottom 96‐well plates, in Xvivo 15 media (Invitrogen) supplemented with 10% human AB serum (Valley Biomedical). After 6 days in culture, proliferation of T cells was assessed by flow cytometry after staining with zombie yellow dye (Biolegend) and CD3‐APC‐Cy7, CD8‐FITC (Tonbo), CD4‐PE, CD103‐PE‐Cy7 (eBiosciences), and CD11c‐PerCp‐Cy5.5 (Biolegend). Naïve T cells alone were used as a negative control. For some experiments, TGFβ receptor 1 blocker, SB431542 (10 μmol/L, Tocris Cookson Inc) (Ochiel, Ochsenbauer, et al., 2010), was added to the cultures at the beginning of each experiment.

### 4.6 Statistics

Data analysis was performed using the GraphPad Prism 5.0 software. A two‐sided *p*‐value <.05 was considered statistically significant. Comparison of two groups was performed with the nonparametric Mann–Whitney *U*‐test or Wilcoxon paired test. Comparison of three or more groups was performed applying the nonparametric Kruskal–Wallis followed by Dunn's post‐test. Correlation analyses were performed applying nonparametric Spearman test. Data are represented as the mean ± *SEM*.

## CONFLICT OF INTEREST

The authors declared no conflict of interest.

## AUTHORS’ CONTRIBUTIONS

MR‐G designed the research; MR‐G, JMF, and FB conducted experiments and acquired data; MR‐G, JMF, and CRW analyzed data; MR‐G and CRW wrote the manuscript.
